# Biochemical characterization of aspartylglucosaminidase missense variants of unclear significance reveals different degrees of functional impairment

**DOI:** 10.3389/fchem.2026.1860625

**Published:** 2026-07-07

**Authors:** Antje Banning, Camille E. Kreuder, Nicara C. Parr, Tabea Lause, Leonie Voll, Halen Godany, Diana Ballhausen, Ritva Tikkanen

**Affiliations:** 1 Institute of Biochemistry, Faculty of Medicine, University of Giessen, Giessen, Germany; 2 Pediatric Metabolic Unit, Pediatrics, Woman-Mother-Child Department, Lausanne University Hospital and University of Lausanne, Lausanne, Switzerland

**Keywords:** lysosomal storage disorder, protein folding, protein stability, protein structure, variants of unclear significance

## Abstract

Prediction of the molecular consequences of missense variants in genetic diseases places a challenge, often resulting in ambiguous classifications by different algorithms. Thus, such variants of unclear significance (VUS) require a thorough molecular characterization to judge if they are pathogenic or not. Pathogenic variants in the *AGA* gene encoding for aspartylglucosaminidase (AGA) result in aspartylglucosaminuria (AGU), a lysosomal storage disorder. For a correct diagnosis, it is important to understand the effect of VUS found in AGU patients on the structure and function of the AGA enzyme. In this study, several *AGA* missense variants found in AGU patients were characterized by biochemical and bioinformatics methods. The pathogenicity of most of these variants was verified, but some of them exhibited only a minor pathogenic effect on AGA function. Especially the Leu126Val variant showed a high residual AGA activity, and a homozygous patient with this variant has a mild AGU phenotype with atypical symptoms. In the era of personalized medicine, our data stress the importance of molecular characterization of unclear genetic variants for the choice of best treatment options.

## 1 Introduction

Aspartylglucosaminuria (AGU, OMIM 208400) is a rare, recessively inherited disease that belongs to the group of lysosomal storage diseases. It is caused by a deficiency of the enzyme aspartylglucosaminidase (AGA; N4-(β-N-acetylglucosaminyl)-L-asparaginase; EC 3.5.1.26). The disorder results in an accumulation of uncleaved glycoasparagines in the lysosomes. The clinical symptoms evolve over time, but the progress of the disease is slower than in many other lysosomal diseases [reviewed in Arvio and Mononen (2016); Goodspeed et al. (2021)]. The spectrum of disease-causing *AGA* gene variants is wide, ranging from deletions and insertions to missense and nonsense point mutations (Saarela et al., 2001). Overall, missense mutations are responsible for the majority of AGU cases. Due to a founder effect, AGU incidence is highest in Finland, with one specific allele (AGU_Fin-major_) found in 98% of the Finnish AGU patients (Fisher and Aronson, 1991; Ikonen et al., 1991a; Mononen et al., 1991). In the AGU_Fin-major_ allele, the exchange of Cys163 to Ser prevents the formation of an intramolecular disulfide bond, whereas the Arg161Gln exchange in the same allele has no pathogenic relevance (Ikonen et al., 1991b; Riikonen et al., 1994; Riikonen et al., 1996). Patients of non-Finnish origin usually exhibit their own family-specific variants that may be compound heterozygous, or homozygous in case of consanguinity (Saarela et al., 2001). When looking at absolute numbers that are available from sequencing projects, some of the non-Finnish variants seem to be as common as, or even more common than the AGU_Fin-major_, while other variants have very low allele counts (https://gnomad.broadinstitute.org/gene/ENSG00000038002?dataset=gnomad_r4). In terms of geographical distribution, it is striking that the more frequent AGA-variants are concentrated in certain regions/ethnicities. For example, AGU_Fin-major_ is present in patients from Finland or in patients of Finnish origin, while the Leu126Val variant seems to have an African or admixed American ancestry, even though it is also found in some other populations (Table 1). The Arg265His variant has the highest allele count in the South Asian genetic ancestry group. In general, one healthy *AGA* allele is sufficient to prevent the disease, and the carriers of one pathogenic *AGA* variant do not develop any symptoms.

**TABLE 1:** Assessment of AGA genetic variants.

Variant	Database entries	Clinical significance (ClinVar)	Remarks on current patients and further references; gnomAD population with highest allele frequency
c.83C>T; p.P28L	ClinVar: no entry dbSNP: rs755612270 gnomAD: 4-177442293-G-A	not reported	Compound heterozygous with frameshift variant (United States of America); European (non-Finnish) allele frequency 0.000001695
c.128–2 A>G	ClinVar: VCV001071135.10 dbSNP: rs2111022003 gnomAD: no entry	pathogenic	Homozygous in a patient of Indian origin (Banning and Tikkanen, 2021)
c.167C>T; p.A56V	ClinVar: VCV001507192.4 dbSNP: rs758886928 gnomAD: 4-177440387-G-A	uncertain significance	Described in (Selvanathan et al., 2021); European (non-Finnish) allele frequency 0.00003136
c.202C>T; p.Q68*	ClinVar: VCV001341346.1 dbSNP: rs1560950756 gnomAD: no entry	pathogenic	Homozygous in a patient of Indian origin
c.214 T>C; p.S72P	ClinVar: VCV000000229.10 dbSNP: rs121964909 gnomAD: no entry	pathogenic	Compound heterozygous with p.W168* (France) (Banning et al., 2018); Further references (Saarela et al., 2001; Peltola et al., 1996; Zlotogora et al., 1997)
c.280G>C; p.G94R	ClinVar: VCV002068004.1 dbSNP: rs758907500 gnomAD: 4-177440274-C-G	uncertain significance	Compound heterozygous with p.W168* (Portugal); European (non-Finnish) allele frequency 0.00001949
c.302C>T; p.A101V	ClinVar VCV000000223.39, dbSNP rs121964908 gnomAD: 4-177439668-G-A	pathogenic/likely pathogenic	Homozygous in Turkish families in Germany; further references (Ikonen et al., 1991c; Park et al., 1993; Saarela et al., 2001); South Asian allele frequency 0.00004393
c.319C>T p.R107*	ClinVar: VCV000291257.17 dbSNP: rs765070743 gnomAD: 4-177439651-G-A	pathogenic	Compound heteroz. with p.E340* in a patient of African origin; homoz. in India; homoz. in China (Gao et al., 2023); African/African American allele frequency 0.00002672
c.347G>A; p.R116Q	ClinVar: no entry dbSNP: rs774967157 gnomAD: 4-177439623-C-T	not reported	Compound heterozygous with AGU_Fin-major_ (Finland); European allele frequency 0.00003128
c.346C>T; p.R116W	ClinVar: VCV000055942.21 dbSNP: rs386833423 gnomAD: 4-177439624-G-A	pathogenic/likely pathogenic	Homozygous in Turkish families in Germany (Opladen et al., 2014); European (non-Finnish) allele frequency 0.000005087
c.365C>A; p.T122K	ClinVar: VCV000557564.42 dbSNP: rs771563230 gnomAD: 4-177439605-G-T	pathogenic/likely pathogenic	Compound heterozygous with large genomic deletion (United States of America) (Banning et al., 2016); European (non-Finnish) allele frequency 0.000002543
c.376C>G; p.L126V	ClinVar: VCV000650146.10 dbSNP: rs200420067 gnomAD: 4-177439594-G-C	uncertain significance	Homoz. in a patient of Algerian origin; admixed American, middle Eastern and African/African American allele frequency 0.001241–0.0017
c.409C>T; p.Q137*	ClinVar: no entry dbSNP: no entry gnomAD: no entry	not reported	Compound heterozygous with AGU_Fin-major_ (Finland)
c.488G>C; p.C163S	ClinVar: VCV000000219.22 dbSNP: rs121964904 gnomAD: 4-177438764-C-G	pathogenic	AGU_Fin-major_, C163S only found together with p.R161Q in *cis* (Fisher and Aronson, 1991; Ikonen et al., 1991a; Mononen et al., 1991); European (Finnish) allele frequency 0.007673
c.503G>A; p.W168*	ClinVar: VCV000055949.13 dbSNP: rs386833430 gnomAD: 4-177438749-C-T	pathogenic/likely pathogenic	Compound heterozygous in several patients (Saarela et al., 2001; Banning et al., 2018); European (non-Finnish) allele frequency 0.000005249
c.535 T>C; p.C179R	ClinVar: VCV000900309.4 dbSNP: rs1187962299 gnomAD: no entry	uncertain significance	Homozygous siblings (Italy)
c.620 T>C; p.I207T	ClinVar: VCV002199163.1 dbSNP: rs772248515 gnomAD: 4-177437407-A-G	uncertain significance	Homozygous (Portugal); middle Eastern allele frequency 0.0001652
c.722C>A p.P241H	ClinVar: VCV002664009.1 dbSNP: rs140889732 gnomAD: no entry	uncertain significance	Compound heterozygous with p.W168* (Germany)
c.794G>A; p.R265H	ClinVar: VCV000348230.11 dbSNP: rs375663828 gnomAD: 4-177434394-C-T	uncertain significance	Described in (Selvanathan et al., 2021); South Asian allele frequency 0.001252
c.904G>A; p.G302R	ClinVar: VCV000000220.1 dbSNP: rs121964905 gnomAD: 4-177433250-C-T	pathogenic	Homozygous in Turkish families in Germany, see also (Ikonen et al., 1991c); European (non-Finnish) allele frequency 0.000001695
c.1018G>T; p.E340*	ClinVar: VCV001879068.3 dbSNP: rs1736641565 gnomAD: no entry	pathogenic/likely pathogenic	Compound heterozygous with p.R107* in a patient of African origin

AGA is synthesized as a 346 amino acid, single-chain precursor molecule that requires an intramolecular autocatalytic processing to form the mature enzyme (Ikonen et al., 1991a). The 23 amino acid long signal peptide is cleaved co-translationally in the ER, where two precursor molecules dimerize and undergo an autocatalytic cleavage between amino acids 205 and 206 (Tikkanen et al., 1996). The active tetrameric enzymes are composed of two alpha and two beta subunits, which receive mannose-6-phosphate (M6P) modifications in the Golgi, and then enter the endolysosomal system in an M6P-dependent way (Tikkanen et al., 1997). Interestingly, most disease-causing AGA variants do not affect the active center of the enzyme, but prevent the correct folding or processing into the two subunits.

In the 1990s, the few AGU variants known back then were compared in terms of activity and processing, and classified according to their severity (Ikonen et al., 1991c; Saarela et al., 2001). In the recent years, due to improvement of genome and exome sequencing techniques, numerous AGU patients with previously unknown gene variants have been identified. Despite current efforts, in many parts of the world, AGU may still be a largely underdiagnosed disease, and reliable incidence values only exist for Finland. To date, the ClinVar database (https://www.ncbi.nlm.nih.gov/clinvar) lists 25 nonsense, 29 splice-site and 151 missense variants of *AGA* that are distributed over the entire coding region, but their interpretation is largely inconclusive. So far, only 10 of the 151 known missense variants have received the classification “pathogenic”, although AGU patients with further listed variants exist. For most of the identified *AGA* gene variants, studies characterizing the consequences of the respective mutated AGA enzymes have not been carried out, and the majority of missense variants are classified as “variants of unknown significance”, or VUS (113 variants in ClinVar) or “likely pathogenic” (8 variants in ClinVar). In addition, the gnomAD browser currently lists 332 *AGA* missense variants that were identified in various genomic sequencing projects but lack any classification, and they are not present in known AGU patients.

A functional validation to classify all *AGA* VUS listed in the databases is challenging. Thus, we have focused our efforts on *AGA* variants found in patients known to us, where we either have primary skin fibroblasts in our biobank, or are in direct contact with the family or the treating physicians. Furthermore, potentially pathogenic VUS found in AGU patients that were described in the recent literature were also addressed. Our data show that most of the investigated *AGA* variants are pathogenic, and are, in terms of enzyme activity and processing, similar to the AGU_Fin-major_ variant. However, some of the patients with specific variants appear to have a higher residual enzyme activity or even a milder phenotype. We propose that a comprehensive characterization of the underlying variant should be carried out for all AGU patients with novel or uncharacterized missense variants. This information is of major importance when deciding on a treatment and when interpreting the success of a therapy. The present comparative study will contribute to a better understanding of the currently prevalent *AGA* variants.

## 2 Results

### 2.1 AGA variants characterized in the present study

**FIGURE 1:**
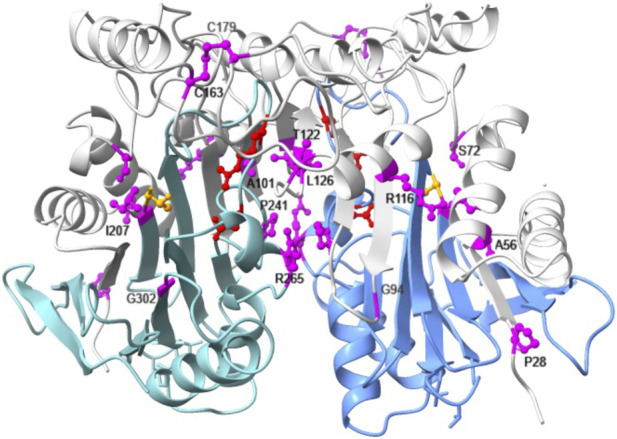
AGA protein structure with the residues altered in the studied variants. The structure of human tetrameric AGA (https://doi.org/10.2210/pdb1APY/pdb) was analyzed in ChimeraX (Meng et al., 2023). The positions of the analyzed missense variants are shown in magenta. The active site Thr206 is indicated in yellow, whereas the substrate binding site residues are depicted in red. The positions of the variants are depicted in one tetramer half for clarity.

For a comprehensive molecular characterization of *AGA* VUS, we chose a total of 21 *AGA* variants (15 missense, five nonsense, one splice site; Table 1). Except for the A56V and R265H variants that were recently described in an Australian hematopoietic stem cell transplantation (HSCT) study (Selvanathan et al., 2021), the variants were selected based on our direct contacts with either the patient families, treating physicians, or patient organizations. Even though AGU has so far been considered as a Finnish heritage disease, the distribution of the variants examined in the present study shows that there are also various non-Finnish AGU patients all over the globe, with mutations along the entire coding region of the AGA gene. Figure 1 shows the localization of the missense variants addressed in this study in the 3-dimensional structure of the AGA enzyme. Almost all variants (except Q137*) are already described in public databases (Table 1), but some of the variants are found in only one of the three databases consulted (ClinVar, gnomAD, or dbSNP database). When searching for information on a patient variant after a genetic diagnosis, it is therefore not sufficient to search in just one database. The individual variants differ to a large degree in terms of their frequency and genetic ancestry (Table 1). Among those examined here, the variants L126V and R265H seem to exhibit a similar frequency as the AGU_Fin-major_ variant that has so far been considered as the worldwide most common AGU-causing variant.

### 2.2 Biochemical characterization of the AGA variants

**FIGURE 2:**
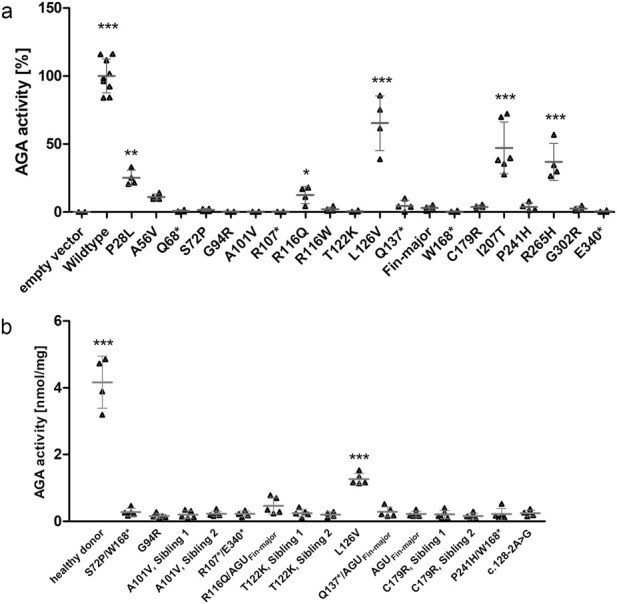
Residual AGA activity in cell lysates. **(a)** AGA knockout HEK293T cells were transiently transfected with the AGA variants, and AGA activity was measured in cell lysates. The activity of wildtype AGA was set as 100%, and the other values were normalized to it. N ≥ 3. **p < 0.01, ***p < 0.001 vs. AGU_Fin-major_ and empty vector. **(b)** Primary skin fibroblasts from 15 AGU patients and one healthy donor were used for AGA activity measurements. Patients with identical AGA variants are siblings. Enzyme activity is expressed as nmol converted substrate per mg of total protein. N ≥ 3. ***p < 0.001 vs. AGU_Fin-major_.

To determine the residual enzyme activity of the individual AGA missense and nonsense variants, expression constructs were produced by mutagenesis PCR and transiently transfected into AGA knockout HEK293T cells. None of these variants reached the activity of the reference wildtype AGA, but several variants, i.e., P28L, R116Q, L126V, I207T and R265H, showed a clearly measurable residual AGA activity that was significantly higher than that of the AGU_Fin-major_ variant (Figure 2a). For the A56V variant, this difference was not significant. Among the variants with significant residual AGA activity, L126V, I207T and R265H exhibited residual activities that reached the levels of about 50% of the wildtype activity, corresponding to values expected for AGU carriers. The investigated nonsense variants (Q68*, R107*, Q137*, W168*, and E340*) showed very little or undetectable AGA enzyme activities.

Primary dermal fibroblasts from several AGU patients, including a patient homozygous for the L126V variant and a patient heterozygous for R116Q and AGU_Fin-major_ variants, were available in the biobank of our laboratory. Fibroblasts of these patients, as well as further ones available in our biobank, were used for AGA activity measurements (Figure 2b). Unfortunately, patient fibroblasts with the variants P28L, I207T, and R265H, which showed high residual activity when overexpressed *in vitro* (Figure 2a), were not available. The fibroblasts of the patient with the AGA variant L126V showed a significant residual AGA activity, whereas the AGA activities in the other available patient fibroblasts exhibited activities comparable to a patient homozygous for the AGU_Fin-major_ variant (Figure 2b). Some of the patients whose fibroblasts were studied are compound heterozygous, including the patient with the R116Q variant. Thus, it may be difficult to interpret the severity of their individual variants, as both alleles contribute to the enzyme activity. However, for the patients who are homozygous for their AGA variants, residual enzyme activities in the patient cells confirmed the results of our overexpression system.

**FIGURE 3:**
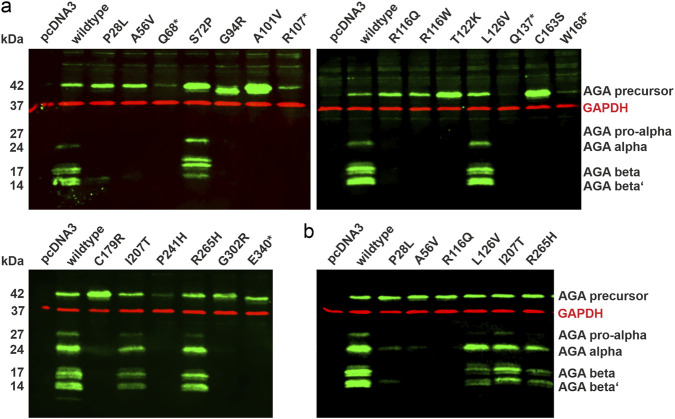
Expression and processing of AGA variants. **(a)** Equal amounts of AGA variants or empty pcDNA3 vector were transiently overexpressed in AGA knockout HEK293T cells, and AGA (green) and GAPDH (red) were detected by Western blotting. **(b)** Processing of the AGA missense variants that displayed a higher degree of residual AGA activity, in comparison to wildtype AGA.

The samples that were examined for AGA activity were also analyzed by Western blot (Figure 3). For AGA to be enzymatically active, two precursor molecules need to dimerize, and are subsequently processed into alpha and beta subunits (Tikkanen et al., 1996). Due to N-glycosylation and further processing steps within lysosomes, active wildtype AGA shows a typical pattern of signals in Western blot, with both the precursor (42 kDa) and the various forms of the subunits, ranging between 27 and 14 kDa (Figure 3). The AGU_Fin-major_ variant is not processed into subunits and exists as an inactive precursor molecule, as already shown in the literature (Banning et al., 2016; Ikonen et al., 1991b). Consistently, the AGU_Fin-major_ variant exhibited only the precursor signal at 42 kDa. The same was true for the low activity missense variants G94R, A101V, R116W, T122K, C179R, and G302R (Figure 3a). Only low amounts or no AGA precursor was found for P241H and the nonsense variants Q68*, R107*, Q137*, and W168*. However, the nonsense variant E340*, whose nonsense codon is located only six triplets upstream of the actual stop codon, exhibited substantial amounts of the precursor polypeptide that was not processed into subunits. The S72P missense variant was aberrantly processed into subunits, as already described (Banning et al., 2018; Peltola et al., 1996). The variants L126V, I207T and R265H showed a processing comparable to the wildtype, consistent with their high residual AGA activity. Other variants that showed a low/intermediate enzyme activity (P28L, A56V, R116Q) exhibited only faint signals for the processed forms, so that the subunits could not be detected in a quantitative way (Figure 3b). This indicates that AGA activity measurements are of higher sensitivity, especially for variants with rather low residual activity.

### 2.3 Bioinformatics analysis of the AGA variants

In addition to our wet-lab experiments, we also investigated the AGA variants using artificial intelligence-based tools (Table 2). The AlphaMissense tool (Cheng et al., 2023) predicts the probability of any variant being pathogenic, by assigning a score between 0–1. In general, variants with a score between 0 and 0.34 are considered to be likely benign, while scores between 0.34 and 0.564 are uncertain, and scores >0.564 classify the variants as likely pathogenic. As a control, we included the AGA neutral variant S149T (Banning et al., 2017) that has a comparable activity as the wildtype AGA and a very low pathogenicity score (Table 2).

**TABLE 2:** AlphaMissense prediction and putative effects of the amino acid substitution. * Categories according to ACMG guidelines, see Richards et al. (2015). See 4.8. For details.

AGA missense variant	AlphaMissense pathogenicity score (0–1)		Change in protein stability (ΔΔG) kcal/mol	Experimental results		Our classification according to ACMG guidelines*	Putative function of this amino acid and/or potential effect of mutation
				Western blot	Activity (%)		
c.83C>T; p.P28L	0.278	likely benign	−0.41 (neutral)	reduced processing	20%–30% (HEK)	pathogenic IIIb (PS3, PM2, PM3, PP2, PP4, BP4)	Close to signal peptide, effect on signal peptide recognition?
c.167C>T; p.A56V	0.375	uncertain	−1.89 (destabilizing)	reduced processing	10%–15% (HEK)	likely pathogenic II (PS3, PM2, PP2, PP5)	Located in alpha-helix, important for stability?
c.214 T>C; p.S72P	0.730	likely pathogenic	−0.11 (neutral)	wrong processing	<5% (HEK) 8% (Fib)	pathogenic IIIb (PS3, PM2, PM3, PP2, PP3, PP4, PP5)	Located in alpha-helix, in vicinity of active site
c.280G>C; p.G94R	0.816	likely pathogenic	−1.17 (destabilizing)	no processing	<5% (HEK and Fib)	pathogenic IIIb (PS3, PM2, PM3, PP2, PP3, PP4)	Close to Asp237 substrate binding site, effect on substrate binding?
c.302C>T; p.A101V	0.597	likely pathogenic	−0.67 (destabilizing)	no processing	<5% (HEK) 6% (Fib)	pathogenic IIIc (PS3, PM2, PP1, PP2, PP3, PP4, PP5)	Located in a beta-strand in the inner part of AGA, effect on substrate binding and enzyme activity?
c.347G>A; p.R116Q	0.307	likely benign	−0.59 (destabilizing)	reduced processing	10%–20% (HEK) 12% (Fib)	pathogenic IIIa (PS3, PM2, PM3, PM5, PP2, PP4)	Located in alpha-helix, can form several hydrogen bonds, important for stability?
c.346C>T; p.R116W	0.183	likely benign	−0.53 (destabilizing)	no processing	<5% (HEK)	pathogenic IIIb (PS3, PM2, PM5, PP2, PP5)	Located in alpha-helix, can form several hydrogen bonds, important for stability?
c.365C>A; p.T122K	0.904	likely pathogenic	−0.39 (neutral)	no processing	<5% (HEK) 6% (Fib)	pathogenic IIIb (PS3, PM2, PM3, PP2, PP4, PP5)	Located on the interface between two αβ dimers, important for stability?
c.376C>G; p.L126V	0.137	likely benign	−1.07 (destabilizing)	normal processing	>50% (HEK) 31% (Fib)	likely pathogenic II (PS3, PM1, PP2)	Located in the inner part of AGA, effect on substrate binding and enzyme activity?
c.488G>C; p.C163S	0.958	likely pathogenic	−1.69 (destabilizing)	no processing	<5% (HEK) 6% (Fib)	pathogenic II (PS3, PS4, PM2, PM3, PP1, PP2, PP3, PP4, PP5)	Forms disulfide bond with C179, important for correct folding
c.535 T>C; p.C179R	0.811	likely pathogenic	−1.15 (destabilizing)	no processing	<5% (HEK) 5% (Fib)	pathogenic IIIc (PS3, PM2, PP1, PP2, PP3, PP4)	Forms disulfide bond with C163, important for correct folding
c.620 T>C; p.I207T	0.339	likely benign	−2.34 (destabilizing)	normal processing	35%–45% (HEK)	VUS - not enough evidence (PM2, PP2, PP4)	Next to active site T206, effect on enzyme activity? May alter splicing
c.722C>A; p.P241H	0.708	likely pathogenic	−1.42 (destabilizing)	no processing	<5% (HEK) <5% (Fib)	pathogenic IIIb (PS3, PM2, PM3, PP2, PP3, PP4)	Oriented towards inner part of enzyme, important for stability?
c.794G>A; p.R265H	0.262	likely benign	−1.45 (destabilizing)	normal processing	30%–40% (HEK)	VUS - not enough evidence (PM3, PP1, PP2, PP5, BS1)	Oriented towards inner part of enzyme, important for stability?
c.904G>A; p.G302R	0.884	likely pathogenic	−0.07 (neutral)	no processing	<5% (HEK)	pathogenic IIIc (PS3, PM2, PP1, PP2, PP4, PP5)	Last residue of a beta-strand, important for correct folding?

**FIGURE 4:**
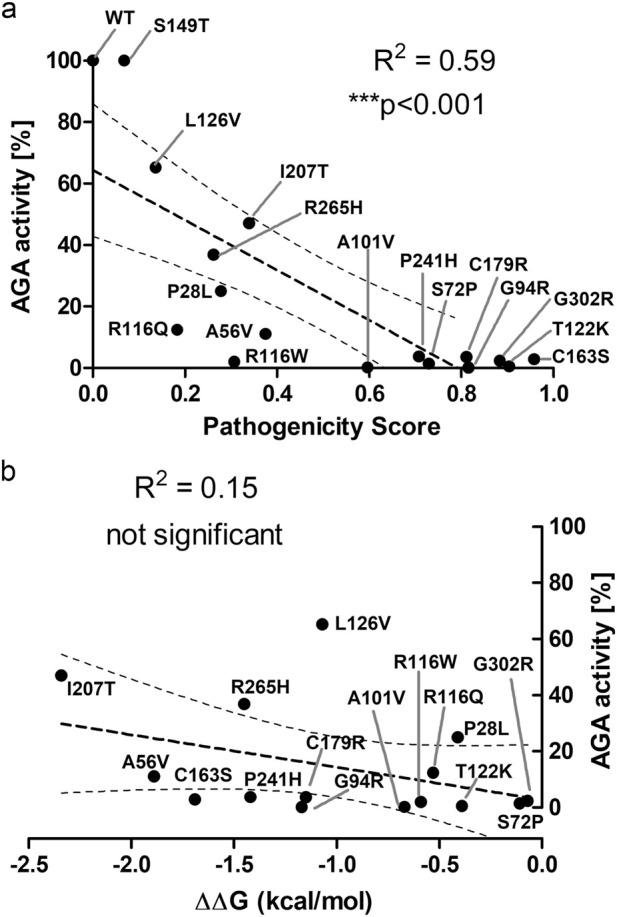
Correlation of the residual AGA activity with the predicted effects on pathogenicity and stability. **(a)** Mean residual AGA activities of the missense variants were correlated with the AlphaMissense (Cheng et al., 2023) pathogenicity scores from Table 2. A significant correlation was observed. **(b)** Mean residual AGA activities were correlated with the protein stability changes predicted by Dynamut2. All missense variants are expected to lead to a destabilization of the AGA enzyme. No significant correlation was found.

When correlating the AlphaMissense scores with the residual AGA activities, a highly significant correlation was found (Figure 4a). Especially the variants without detectable enzyme activity had AlphaMissense scores close to 1, while the L126V variant with the highest residual activity had a very low score. An exception to the significant correlation was the variant R116W with undetectable enzyme activity, but with a low/moderate pathogenicity score, whereas the variants that showed a moderate enzyme activity were also assigned a moderate pathogenicity score. Overall, the AlphaMissense tool can provide useful information, but it cannot replace biochemical analysis.

The Dynamut2 webtool assesses the impact of missense variants on protein stability by calculating the change in Gibbs free energy ΔΔG, i.e., the difference between ΔG of mutated and reference protein. A negative value points to a decrease in stability in comparison to the reference protein. No official threshold values exist, but commonly, values between −0.5<ΔΔG<0.5 kcal/mol are considered neutral (Hossain et al., 2025; May et al., 2025). In contrast to the pathogenicity scores (Figure 4a), prediction of protein stability changes with the Dynamut2 tool did not result in conclusive data, as all investigated variants were predicted to lead to protein destabilization of varying degrees (negative ΔΔG values) that was considered neutral for the S72P, T122K and G302R variants. However, there was no correlation between the observed residual activities and the predicted protein stability changes (Table 2; Figure 4b). The S149T variant with 100% AGA activity was not included in this analysis, as we have previously shown that it does not affect the stability of the AGA polypeptide (Banning et al., 2017).

**FIGURE 5:**
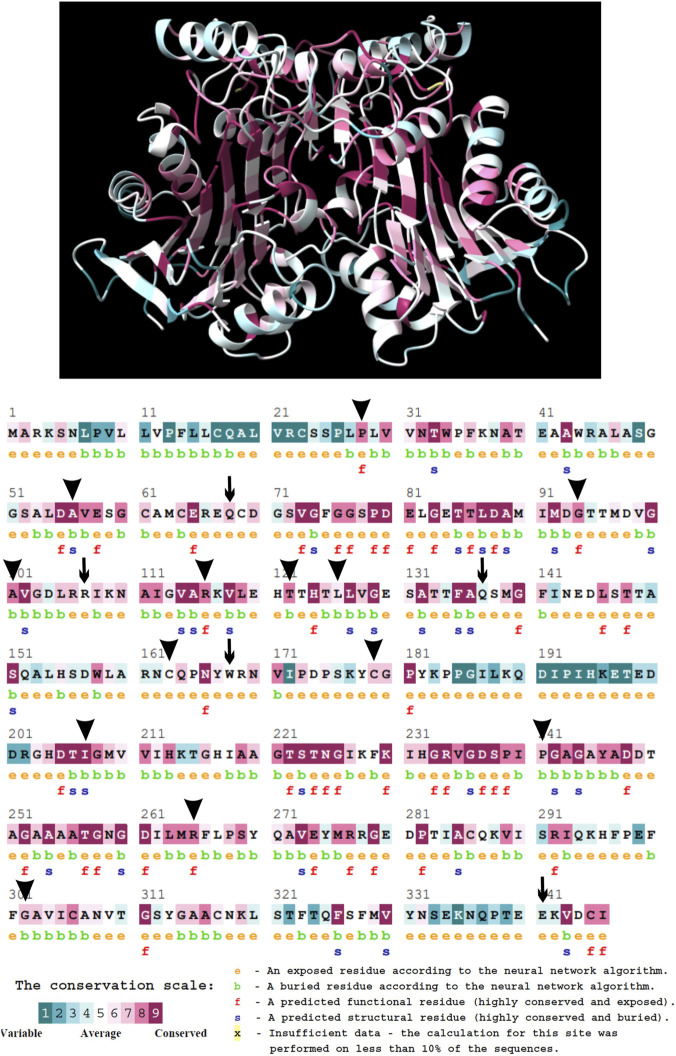
Spatial conservation analysis of AGA using a multi-species orthologue comparison. For the spatial conservation analysis, the human AGA structure was analyzed *via* ConSurf-DB (Ben Chorin et al., 2020), by which the sequence is automatically compared to multiple homolog structures from the UniProt database. Slim arrows indicate the positions of the nonsense variants, whereas thick arrowheads indicate the residues of the missense variants analyzed in this study. All missense variants, except L126V and AGU_Fin-major_ variants, replace conserved amino acids.

Since correct processing into subunits is a prerequisite for an active AGA enzyme, a general prediction of protein stability in the variants does not appear meaningful. Most pathogenic AGA variants affect conserved amino acids (Figure 5), and spatial conservation analysis of AGA with a multi-species orthologue comparison showed that, among the amino acids altered in the missense variants, only L126, and C163 of the AGU_Fin-major_ variant are evolutionarily not conserved.

### 2.4 Lysosomal enrichment and the presence of selected variants in the lysosomes

**FIGURE 6:**
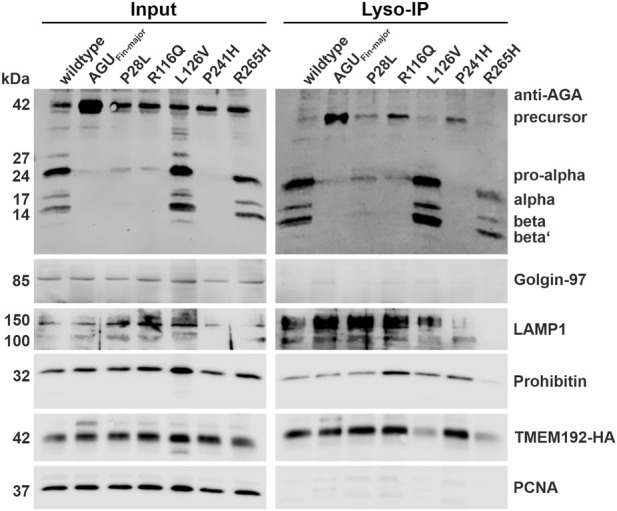
Lyso-IP for the enrichment of lysosomes. AGA wildtype and the variants were transiently transfected in AGA knockout cells, in which the endogenous TMEM192 carried an HA tag. Lyso-IPs were compared to input samples. Success of the lysosomal enrichment was verified with different organelle markers.

AGA is a lysosomal enzyme that travels through the secretory biosynthesis pathway and enters the lysosomes mainly in an M6P-dependent manner (Tikkanen et al., 1995; Tikkanen et al., 1997). Neither the AGA activity measurement nor the Western blots allowed us to determine whether the AGA variants reached the lysosome, or if they were misdirected or retained in the rER or Golgi. We therefore investigated several AGA variants, particularly those with high residual enzyme activity, and the AGU_Fin-major_ variant with Lyso-IPs. With this method, intact lysosomes are enriched by immunoprecipitation of the endogenous lysosomal membrane protein TMEM192 that is tagged with an HA tag (Abu-Remaileh et al., 2017). Wildtype AGA reached the lysosome and was detected in the lysosome-enriched fraction, as were all AGA missense variants examined with this assay (Figure 6). Importantly, processed AGA with the subunits was the major form visible in the lysosomal fraction for the L126V and R265H variants, and it was detectable, together with the precursor form, for the P28L and R116Q variants. However, AGU_Fin-major_ and P241H variants were detected as unprocessed precursors even in the lysosomal fraction. Hence, transport to the lysosome does not seem to require the correct processing and activation of the AGA enzyme, as it also occurs, at least to some degree, with some AGA variants that remain in their precursor state. Although our isolated Lyso-IP fraction is enriched with lysosomes, mitochondrial proteins (prohibitin) are also present. However, a profound missorting or retention in the early secretory compartments of the AGA variants is unlikely.

## 3 Discussion

In this study, we have carried out a molecular characterization of several potentially pathogenic AGA variants, many of which were classified as VUS or received discrepant classifications by the prediction algorithms. Our data show that the pathogenicity scores obtained through AI-based tools, such as Dynamut2, are not always capable of clearly depicting pathogenic missense variants and distinguishing them from the benign ones. Therefore, a more comprehensive molecular characterization, including measurement of the residual enzyme activity, verification of the cellular localization, and assessment of the autocatalytic activation of AGA are required to correctly assign the variants into different classes. The results of our study also stress the importance of a systematic and, whenever possible, experimentally verified classification that should be included in all databases that are used by clinicians for judging patient mutations. Optimally, these verification steps should also involve patient cells.

Overall, we were able to confirm the pathogenicity of the variants Q68*, S72P, A101V, R107*, R116W, T122K, W168*, G302R, and E340* (Table 1), some of which we have already analyzed in our previous studies (Banning et al., 2016; Banning et al., 2018; Banning and Tikkanen, 2021). However, the AGA variants that have previously been classified as VUS (A56V, G94R, L126V, C179R, I207T, P241H, and R265H) or have no previous assignment for their clinical significance (P28L, R116Q, and Q137*) in the databases need to be reclassified, based on our experimental data. The G94R, Q137*, C179R, and P241H variants were now classified as pathogenic according to the ACMG classification criteria (Table 2). They exhibit a very low residual enzyme activity and remain as inactive precursor molecules, so that it is plausible to expect that they are causative for AGU.

**FIGURE 7:**
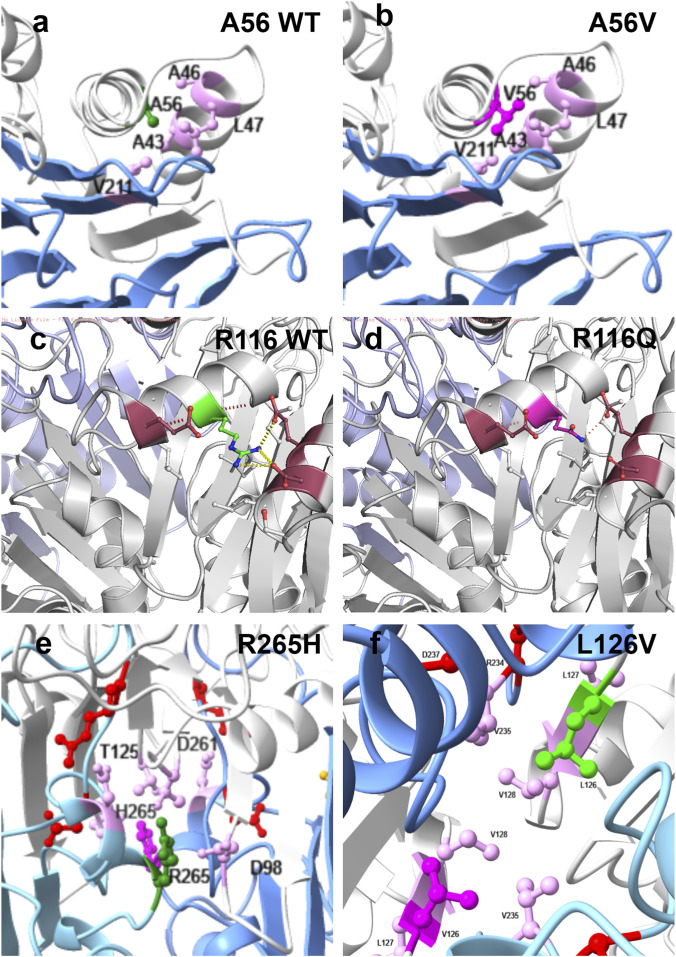
Predicted structural consequences of the AGU variants. DynaMut2 tool (Rodrigues et al., 2018) was used to substitute the individual amino acids of the AGA polypeptide. The resulting AGA structures were visualized using ChimeraX (Meng et al., 2023) **(a,b,e,f)** or PyMOL (**(c,d)**; Schrödinger, LLC, https://www.pymol.org), with the original residues shown in green and the mutated residues in magenta. Residues that are expected to interact with the altered residues are shown as colored ball-sticks. The substrate binding site residues are shown in red.

The variants P28L, A56V and R116Q are pathogenic or likely pathogenic, but their residual AGA activity in our *in vitro* expression system was untypically high for a pathogenic variant. Therefore, there is a slight discrepancy between the experimental data and ACMG classification. For the P28L and A56V variants, fibroblasts from the patients were not available, and the activity measurements would need to be verified in patient cells. P28 is a highly conserved amino acid (Figure 5) located close to the N-terminus of the alpha subunit, being the fifth residue of the mature alpha subunit. Therefore, the P28L substitution could have an effect on the removal of the signal peptide, or it may affect the polypeptide conformation (Table 2). A56 is also highly conserved and predicted to be structurally important (Figure 5). It is located in an α-helix at the interface of two helices, and the bulkier Val residue may impose a structural constraint in this region, impairing the stability (Table 2; Figures 7a,b). Unfortunately, cells or other samples, such as serum, for the patients with the P28L and A56V variants were not available. However, a homozygous male patient with the A56V variant has been described in Selvanathan et al. (Selvanathan et al., 2021), as he received an HSCT at the age of 5 years. Before the HSCT, the biochemical biomarkers (glycoasparagines in the urine and leukocyte AGA activity) were typical for AGU, and the patient showed a moderate intellectual disability (Selvanathan et al., 2021). However, due to the early HSCT, it is not possible to judge if the patient would have shown a developmental regression typical for AGU without HSCT.

R116 is a surface-exposed, highly conserved residue located in an α-helix (Figure 5; Table 2), and it forms a network of ionic interactions with the surrounding negatively charged residues (Figures 7c,d). Loss of the positive charge upon the R116Q substitution is thus likely to impair the structural stability of the AGA polypeptide. The R116Q variant has been reported in European, non-Finnish alleles several times (Table 1), and it is also found in a Finnish female patient heterozygous for the AGU_Fin-major_ variant. In our *in vitro* expression system, the R116Q variant showed significant residual activity, whereas in the patient fibroblasts, the residual activity was measurably, but non-significantly, higher than in fibroblasts homozygous for AGU_Fin-major_. There are no longitudinal data on the development of the Finnish patient, but her phenotype appears not to be clearly different from the classical AGU. Thus, the positive effect of the “mild” R116Q allele may be masked by the AGU_Fin-major_ allele, and the heterozygous combination in the patient affects the residual enzyme activity, as AGA heterodimers between the two missense variants are likely to form. In addition to the R116Q substitution, a pathogenic variant c.346C>T; p.R116W has also been described in a consanguineous Turkish family with three siblings with AGU (Opladen et al., 2014). In contrast to the R116Q variant, the R116W substitution, with a loss of the positive charge and constraints generated by the bulky aromatic residue, results in a more profound loss of enzyme function (Banning et al., 2016), indicating that the position 116 in the AGA polypeptide chain may be especially sensitive to changes.

Two of the missense variants (I207T and R265H), previously classified as VUS, remained in this category after the ACMG classification, despite our molecular characterization. Our *in vitro* analysis showed that the I207T variant is comparable to the wildtype AGA in terms of precursor processing, and it showed almost 50% residual enzyme activity. However, a Portuguese patient homozygous for the variant has a confirmed diagnosis of AGU, with the typical clinical features and biomarkers of the disease, suggesting that the variant is indeed pathogenic. In the AGA structure, Ile207, a highly conserved amino acid (Figure 5), is the second amino acid of the beta subunit, immediately following the catalytic Thr206. Thus, it is possible that there is a structural effect that impairs the catalysis or the enzyme stability, even though I207T was classified by AlphaMissense analysis as “likely benign”, based on its low pathogenicity score (Table 2). However, the genetic variant, i.e., nucleotide exchange c.620 T>C, resides very close to an exon-intron border, as the third-last base of the exon, so that it may alter the splicing of the AGA pre-mRNA. The potential effect on splicing, which would explain the pathogenicity of this variant, should be studied in patient cells, but they were not available for our studies.

The R265H substitution results in a loss of the positive charge and changes the character of the residue due to the imidazole ring structure in the histidine residue. The highly conserved R265 is located at the interface of two αβ dimers, and is likely to be important for the stability of the active tetrameric enzyme (Figure 7e). The R265H variant has been described in an Iranian Mandean family with highly consanguineous parents (Selvanathan et al., 2021). Due to previous AGU cases in the family, one of the children was diagnosed as a neonate, but was heterozygous with the R265H variant and a splicing defect, despite the consanguineous origin. The AGU biomarkers showed elevated glycoasparagines and reduced AGA activity, confirming the diagnosis. Due to the early diagnosis, the patient was subjected to HSCT at 5 months of age, and 7 years after the transplantation, he shows normal leukocyte AGA activity, and his urine glycoasparagines are very low. He has only minor mental deficits, and attends a normal school with support (Selvanathan et al., 2021). As there are no longitudinal developmental assessments prior to the HSCT, it is not possible to judge if the apparently mild R265H variant contributes to the current mild phenotype of the patient, or if it was rather caused by the HSCT at young age. Thus, one should be careful with interpreting this solely as a result of the HSCT.

We would classify the L126V variant as likely pathogenic according to the ACMG guidelines, even though this variant shows a normal processing and the highest residual activity of all AGU variants studied by us. Even though L126 is not a conserved amino acid (Figure 5), it is buried within the AGA structure and forms a network of hydrophobic interactions with Val and Leu residues from both tetramer halves (Figure 7f). An impairment of these interactions may moderately destabilize the tetramer or even affect the active site conformation (Table 2). In the fibroblasts of the homozygous male patient with the L126V variant, the residual enzyme activity was 31% of our control cells that show a high AGA activity. This would imply that the patient’s AGA activity is relatively close to a carrier value. The patient has very low but detectable urine glycoasparagine levels that are about 10% of the levels in most AGU patients. Healthy individuals should not have any detectable glycoasparagines in urine. The only other disease in which urine glycoasparagines are observed is the congenital disorder of deglycosylation 1 (NGLY1-CDDG), also known as alacrimia-choreoathetosis-liver dysfunction syndrome, which was excluded by genetic diagnosis. The symptoms of the patient are atypical for AGU, as he lacks the coarse facial features and shows numerous behavioral problems, such as autistic behavior and autoaggression, instead of the typical mental impairment observed in AGU (see methods for a detailed description). Due to the moderate nature of the L126V variant, this phenotype may represent an unexpected, mild AGU variant form that has not been previously described in the literature.

Taken together, we here show that for the classification of potentially disease-causing missense variants, various data including evolutionary conservation, effect of the substitution on protein stability and functional analysis such as activity measurements, should be integrated. However, in the case of AGA, the predictions may not always be capable of revealing all effects of a missense variant, as AGA undergoes an autocatalytic cleavage of the precursor polypeptide into the two subunits. Furthermore, evolutionary conservation that in most cases provides important data on the structurally or functionally important residues may not always identify residues whose exchange may be deleterious, such as Cys residues involved in disulfide bonds or Asn residues that are glycosylated. This is, for example, the case of the Cys163Ser substitution in AGU_Fin-major_, which is not a conserved residue.

The results of our study also have important implications for future therapy of AGU patients. Currently, no approved treatments are available for AGU, but we have recently published a potential pharmacological chaperone therapy using a small molecule, betaine (Banning et al., 2016; Laine et al., 2026). As missense variants that are only mildly impaired are more likely to be amenable to this treatment, a molecular characterization of the patient variants is of great importance. For the emerging but so far only experimental or preclinical treatments, such as gene therapy or enzyme replacement, it is also important to know if the patient has some AGA protein expressed, as this would help to estimate the potential risk of immune reactions against the therapeutic agent. Furthermore, for the assessment of the treatment effect, it is necessary to determine the residual enzyme activity for patients with uncharacterized variants.

## 4 Materials and methods

### 4.1 Cell culture

HEK293T (human embryonic kidney) cells were cultured in Dulbecco’s modified Eagle’s medium (DMEM) with high glucose, 10% fetal bovine serum (FBS), 1% penicillin/streptomycin (all from Thermo Fisher Scientific, Dreieich, Germany). *AGA* knockout HEK293T cells without endogenous AGA expression were created by CRISPR/Cas9 (Kapahnke et al., 2016).

Primary skin fibroblasts from an AGU_Fin-major_ patient were obtained from Coriell Institute of Medical Research (Cat. GM00568; Camden, NJ, United States). Primary skin fibroblasts from AGU patients were either obtained from collaboration partners, or were isolated from skin punch biopsies according to our standard procedures (Banning et al., 2025). Parents of the patients provided a signed informed consent for the biopsy, fibroblast culture, storage, enzymatic and molecular analyses (approval of the ethics committee of the Medical Faculty of the University of Giessen, #144/21). The presence of the diagnosed *AGA* variant was verified by targeted gene sequencing (Microsynth Seqlab, Göttingen, Germany). Fibroblasts were cultured in DMEM (high glucose), 10% FBS, 1% penicillin/streptomycin, 1% non-essential amino acids and 1% sodium pyruvate. As controls, immortalized skin fibroblasts were used (Banning et al., 2016). All cells were grown at 8% CO_2_ and 37°C. Information on all *AGA* variants addressed in this study is summarized in Table 1.

### 4.2 Plasmids and transfections

The AGA-pcDNA3 construct (Banning et al., 2017) was used for transient transfections in *AGA* knockout HEK293T cells and served as template for site-directed mutagenesis to create the *AGA* variants to be investigated. All constructs were verified by sequencing. For transient transfections, cells were seeded onto 12-well plates on the day before transfection, and 500 ng of AGA expression constructs were transfected using MACSfectin™ reagent (Miltenyi Biotec, Bergisch Gladbach, Germany) according to the manufacturer’s protocol. The following day, the cells were transferred onto six or 10 cm dishes and harvested after further 48 h.

### 4.3 AGU patient with L126V variant with an untypical form of AGU

An AGU patient with a c.376C>G; p.L126V variant is the first child of consanguineous parents of Algerian origin. He was born at term, and his psychomotor development was normal until the age of 2 years when he started to show repetitive movements. An autism spectrum disorder was suspected at this time. Significant regression occurred at the age of 3.5 years in the context of the family moving to another place and the birth of a brother. The patient barely talked, showed an altered behavior and isolated himself from any social activities, even within the family. He displayed choleric crises, during which he was auto-aggressive and aggressive to anybody around. These crises were triggered by pain, discomfort or frustration. A whole exome sequencing was performed at this time, with the search term “autism spectrum disorder”, and a homozygous variant c.376C>G; p.L126V in the *AGA* gene was identified. AGA enzyme activity was found to be decreased, but with a considerable residual activity (7.74 nmol/24 h/mg protein, normal range 20.4–55.4). Excretion of aspartylglucosamine in the urine was detectable, but it was about 10 times less than in most AGU patients (Laine et al., 2026). Thus, a diagnosis of a mild/untypical form of AGU was established. The patient integrated into a specialized school. Due to difficulties in falling asleep, a treatment with melatonin was initiated, but was found ineffective. His physiognomy was not typical for AGU, as he did not exhibit macrocephaly, tall stature, the typical coarse facial features, or hepatosplenomegaly. A brain MRI at the age of 6.5 years did not reveal any particularities.

The patient is currently 11 years old and attends a special school. Auto-aggression has worsened with age. His BMI went down to a lower percentile, as he continues to be very selective with food, with long phases of lack of appetite and only few variations in the food that he accepts. He is slightly constipated and goes to the toilette several times per hour to urinate, without an organic cause. He has a strong relationship with his teacher, but he does not participate in the activities with the other children of his class. He shows signs of an obsessive-compulsive disorder with repetitive hand washing, leading to severe eczema of the hands. At home, he sticks to his mother who cannot perform any other activities than caring for him.

### 4.4 AGA enzyme activity measurements

AGA activity in cell lysates was measured fluorometrically. Reaction mixtures consisted of 20 µL sample (blank: lysis buffer) and 20 µL of Asp-AMC (L-Aspartic acid β-(7-amido-4-methylcoumarin); 50 µM in McIlvain’s phosphate-citrate buffer pH 6.5). The samples were incubated for 4 h (overexpressing HEK293T) or for 24 h (fibroblasts) at 37°C, after which the reaction was terminated by adding 200 µL McIlvain’s buffer pH 4.5. All samples were measured in triplicates with a Tecan Infinite M200 plate reader (Tecan, Männedorf, Switzerland) using 355 nm excitation and 460 nm emission wavelengths. For quantification, an AMC standard curve, containing increasing amounts of AMC, ranging from 0 to 100 pmol AMC, was measured in parallel. Because of the background fluorescence of Asp-AMC, a specific concentration of Asp-AMC was added, so that the total amount of AMC and Asp-AMC was identical in all standards (Banning et al., 2023).

### 4.5 Western blot

For analysis of total lysates, cells were grown in 6-well plates or 10 cm dishes, lysed in 50 mM Tris, pH 7.4, 150 mM NaCl, 2 mM EDTA, 1% Nonidet P-40, supplemented with protease inhibitors. Equal protein amounts were analyzed with 15% SDS-polyacrylamide gel electrophoresis and Western blot on a nitrocellulose membrane. Primary antibodies were: rabbit anti-AGA (Tikkanen et al., 1995), mouse anti-GAPDH (Abcam, Cambridge, United Kingdom; #ab-8245), rabbit-anti-HA-tag (Abcam, #ab9110), mouse anti-PCNA (sc-56, Santa Cruz Biotechnology, Heidelberg, Germany), mouse anti-Golgin-97 (A21270, Invitrogen, Thermo Fisher Scientific), rabbit anti-LAMP1 (Abcam, #ab24170), and rabbit anti-prohibitin (EP2803Y, Abcam, #ab75766). Detection was performed with an Odyssey® XF Imaging System (LI-COR Biotechnology, Bad Homburg, Germany).

### 4.6 Immunoprecipitation of lysosomes (Lyso-IP)

For endogenous tagging of the lysosomal protein TMEM192 with 3xHA tag in HEK293T AGA knockout cells, the protocol described by Park et al. was used (https://dx.doi.org/10.17504/protocols.io.4r3l24kxxg1y/v2). In short, a gRNA (gRNA-TMEM192-fwd: 5′-CAC​CGA​GTA​GAA​CGT​GAG​AGG​CTC​A-3’; gRNA-TMEM192-rev: 5′-AAA​CTG​AGC​CTC​TCA​CGT​TCT​ACT​C-3′) targeting a sequence adjacent to the translational termination sequence in TMEM192 was cloned after BbsI digestion into pSpCas9(BB)-2 A-Puro (PX459, Addgene plasmid #48139). The resulting plasmid was cotransfected into to the AGA-deficient cells together with pSMART (Addgene plasmid #175777) that contains 5′ and 3′ homology arms for TMEM192, in which the termination codon was replaced by a 3xHA epitope sequence, followed by a TAA stop codon. Puromycin selection (2 μg/mL) was started after 5 days and was continued for 1 week. Thereafter, the cells were counted and seeded onto 96-well plates (1 cell/well). Wells containing single-cell clones were expanded and used for further analysis. Successful tagging of TMEM192 was confirmed *via* Western blotting with an anti-HA antibody. Lyso-IPs were performed according to the protocol from Abu-Remaileh *et al.* (Abu-Remaileh et al., 2017), with some modifications. AGA knockout TMEM-HA cells were transiently transfected with different AGA expression constructs. One confluent 10 cm dish of cells was used per IP. The cells were pelleted and resuspended in 500 µL of cold potassium-based PBS (KPBS, 136 mM KCl, 10 mM KH_2_PO_4_, pH 7.25) and gently homogenized with 50 strokes of a 2 mL homogenizer. The homogenate was centrifuged at 1,000 *g* for 2 min at 4°C and 350 µL of the supernatant was incubated with 40 μL Pierce™ mouse-anti-HA Magnetic Beads (Thermo Fisher Scientific) on a spinning wheel for 1 h at 4°C. The remaining sample was kept as an input sample. Immunoprecipitates were washed three times with KPBS and pulled down on a magnetic stand. Beads were resuspended in 100 µL lysis buffer and sonicated for 5 s, after which the samples were used for Western blotting.

### 4.7 In silico analysis of protein stability

The X-ray crystal structure of human AGA (Oinonen et al., 1995) (PDB ID: 1APY) was subjected to *in silico* mutagenesis using the DynaMut2 web tool (Rodrigues et al., 2018). Predictions of changes in the protein stability (ΔΔG) were obtained, and protein structures were visualized using UCSF ChimeraX (Meng et al., 2023) or PyMOL Molecular Graphics System (Schrödinger, LLC, https://www.pymol.org/). The AlphaFold protein structure database was used to predict the AlphaMissense pathogenicity score of the individual *AGA* gene variants (Cheng et al., 2023) (https://alphamissense.hegelab.org/search). For spatial conservation analysis, the AGA structure was analyzed *via* ConSurf-DB (Ben Chorin et al., 2020).

### 4.8 Classification of pathogenicity according to ACMG

To classify the AGA missense variants, we made use of the guidelines of the American College of Medical Genetics and Genomics (ACMG). For each variant, the criteria given in Tables 3, 4 of Richards et al. (2015) were considered, and the final classification was made according to the „rules for combining criteria to classify sequence variants“ (Richards et al., 2015). In general, each missense variant was categorized according to different criteria that are ranked according to their evidence in very strong (PVS1), strong (PS1, PS2, PS3, PS4), moderate (PM1, PM2, PM3, PM4, PM5, PM6), supporting (PP1, PP2, PP3, PP4, PP5), stand-alone (BA1), strong (BS1, BS2, BS3, BS4), and supporting (BP1, BP2, BP3, BP4, BP5, BP6, BP7). The online tool developed by Kleinberger et al. was used to facilitate the classification of the variants (Kleinberger et al., 2016).

### 4.9 Statistical analysis

All experiments were performed at least three times. The data are expressed as mean ± SD. Statistical comparisons between groups were made using Student’s t-tests, 1way or 2way ANOVA (Analysis of Variance), as appropriate, using the GraphPad Prism five software (San Diego, CA, United States). Values of *p* < 0.05 were considered significant (*), while values of *p* < 0.01 were considered very significant (**), and *p* < 0.001 extremely significant (***).

## Data Availability

The data presented in the study are deposited in the ClinVar repository, accession numbers SCV007602671, SCV007602669, and SCV007602643.
